# Mechanical bone strength decreases considerably after microwave ablation–Ex-vivo and in-vivo analysis in sheep long bones

**DOI:** 10.1371/journal.pone.0292177

**Published:** 2023-10-12

**Authors:** Hendricus Nijland, Jinwen Zhu, Thomas C. Kwee, Ding-Jun Hao, Paul C. Jutte

**Affiliations:** 1 Department of Orthopaedic Surgery, University Medical Center Groningen, Groningen, The Netherlands; 2 Department of Orthopaedic Surgery, HongHui Medical Center Xi’an, Xi’an, China; 3 Department of Radiology, University Medical Center Groningen, Groningen, The Netherlands; Universidade de Trás-os-Montes e Alto Douro: Universidade de Tras-os-Montes e Alto Douro, PORTUGAL

## Abstract

**Background:**

Bone metastases are on the rise due to longer survival of cancer patients. Local tumor control is required for pain relief. Microwave ablation (MWA) is a technique for minimally invasive local tumor treatment. Tumor tissue is destroyed by application of local hyperthermia to induce necrosis. Given the most common setting of palliative care, it is generally considered beneficial for patients to start mobilizing directly following treatment. No data on mechanical strength in long bones after MWA have been published so far.

**Materials and methods:**

In- and ex-vivo experiments on sheep tibias were performed with MWA in various combinations of settings for time and power. During the in-vivo part sheep were sacrificed one or six weeks after ablation. Mechanical strength was examined with a three-point bending test for ablations in the diaphysis and with an indentation test for ablations in the metaphysis.

**Results:**

MWA does not decrease mechanical strength in the diaphysis. In the metaphysis strength decreased up to 50% six weeks after ablation, which was not seen directly after ablation.

**Conclusion:**

MWA appears to decrease mechanical strength in long bone metaphysis up to 50% after six weeks, however strength remains sufficient for direct mobilization. The time before normal strength is regained after the remodeling phase is not known.

## Introduction

The incidence of bone metastases is on the rise due to improved survival of cancer patients [1]. Local tumor control is required for pain relief. Current practice includes radiation, intralesional curettage and wide resection as potential treatment approaches. Microwave ablation (MWA) is a technique for minimally invasive tumor treatment and is therefore an alternative to current, more invasive forms of treatment. In MWA tumor tissue is destructed by application of local hyperthermia. MWA is frequently used for local control of metastases in organs like liver, kidney and lung [2]. However, the use of MWA in bone is still limited and therefore literature on its working mechanism is scarce. No literature exists on the effect of MWA on bone strength. In bone tumor ablation with Radiofrequency Ablation (RFA) fractures occur in 2.6–4.8% of cases [3, 4]. This is comparable to intralesional curettage (4.0%) and lower than for wide resection (23.1%) [5, 6].

When standing, a load of ‘body mass * 9,81 m/s^2^ (gravity acceleration)’ is put on the legs. For an average patient of 80 kg this comes down to almost 400N load per leg. When walking, this load is only slightly higher. However, this load doubles when running at low speed, is threefold for running at higher speed and even sevenfold for jumping and landing [7–9]. Since there is no literature on the mechanical effects of MWA in long bone it is uncertain whether direct mobilization is safe and what the risk is of early return to full activity.

To ensure safe treatment and more evidence-based practice after MWA treatment the present study was performed to assess the biomechanical effect of local tumor ablation in long bone using MWA in an experimental model. Initial strength directly after ablation was hypothesized to be unchanged as local bone architecture is unchanged. As a result of osteoclast activity and remodeling, weakening of bone strength is hypothesized in the weeks after ablation.

## Method

### Microwave ablation

All ablations were performed with a 2.45GHz Kang-You 2000 microwave ablation generator (Kang-You Medical, Nanjing, China). For mechanical testing three-point bending test and indentation test were used, as described in a later section. For the three-point bending test ablation was carried out in the center of the diaphysis. For the indentation test ablation was performed in the proximal metaphysis, at a depth of 30mm, 25mm distal from the cortex. Both an ex-vivo and an in-vivo study were performed.

### Ex-vivo

Fresh sheep bones were collected from the slaughterhouse on the day of experiments. Bones were from similar size and mass. MWA procedures were performed 6–12 hours after the sheep were sacrificed to ensure good fresh bone quality. First, bones were put into 37°C water (range 37–40°C) for 20–30 minutes to mimic body temperature. Holes for the ablation needle and temperature probe were drilled directly before ablation to prevent water getting inside the bone. Ablation was carried out with four different settings: 60W – 10 min, 60W – 20 min, 80W – 20 min and 80W – 10 min. With these settings the effects of time and wattage could be evaluated separately. Wattage levels were chosen based on pilots and earlier reported results by our group [10]. For every setting six bones were used for both mechanical tests. A slow-cooking algorithm for temperature was used [10]. This consists of slowly increasing temperature towards the aimed temperature to lower the risk of carbonization around the needle to occur. Every ablation started with 10W for one minute, followed by one minute at 20W. These two minutes are included in the time settings as mentioned. After ablation, bones were prepared for mechanical testing.

### In-vivo

**Fig 1:**
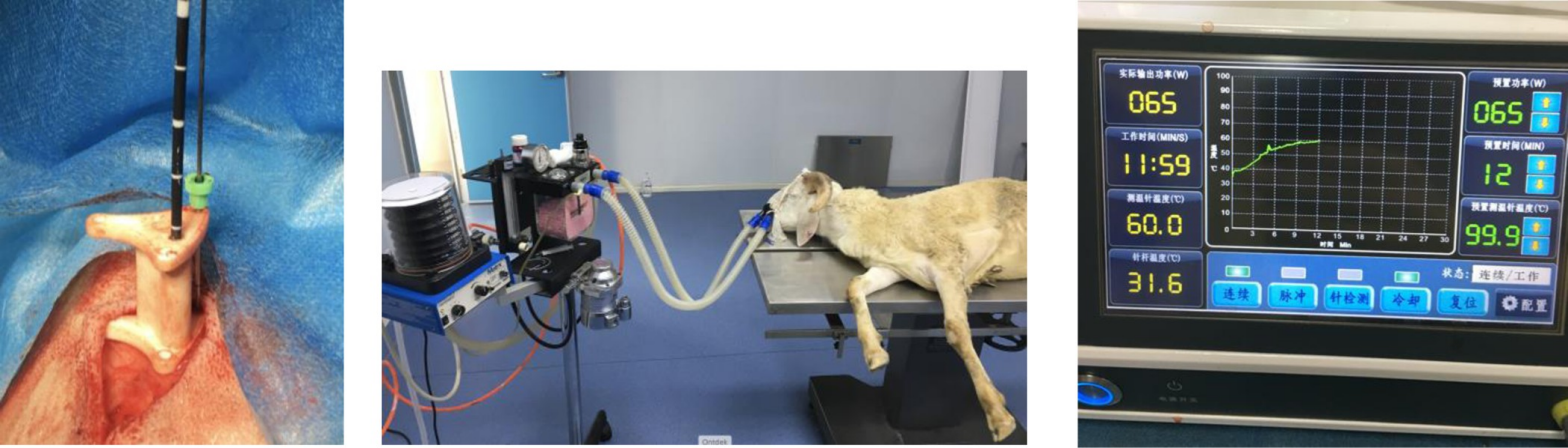
In-vivo procedure. A. Ablation probe (black) and temperature probe inserted in the bone through a positioning device. B. Intubated sheep before procedure. C. the MWA generator.

For the experiment permission was obtained from the medical ethical committee of the HongHui hospital, Xi’an, China. Ablations were performed in tibia of male sheep (Small-tail Han) aged 1–1.5 years with aimed mass of 50 kg (in vivo experiment: range 45–50 kg). Three settings were examined for both a one week- and a six weeks follow-up group: 60W 10min, 60W – 20 min and 80W – 10 min. For each group four sheep were included, leading to a total of 24 sheep. As controls the contralateral tibias were used. Sheep were randomly divided in the one- or six week group. Before starting the procedure (Fig 1A), the sheep were sedated with an intramuscular injection of 5 ml Shutai (1: 1 combination of Tiletamine 50mg/ml & Zolazepam hydrochloride 50mg/ml) (Virbac, France) and subsequently intubated (Fig 1B. During the complete procedure inhaled isoflurane (1–5%) was used for general anesthesia and a maintenance dose up to 2.5ml Shutai was given in case deemed necessary. For post-procedural analgesia meloxicam injections were used. After the follow-up period sheep were euthanized by an experienced veterinarian (bleed out under intramuscular sedation). Sheep with a (deep) infection or fracture were excluded from mechanical analysis to prevent finding differences in strength based on other factors than the mechanical effect of MWA on bone.

### Mechanical testing

For the ex-vivo experiments both indentation test and three-point bending test were performed. These are both generally used tests [11, 12]. To limit the number of animals needed, only indentation test was performed in the in-vivo experiments after analyzing the ex-vivo results.

#### Three-point bending test

**Fig 2:**
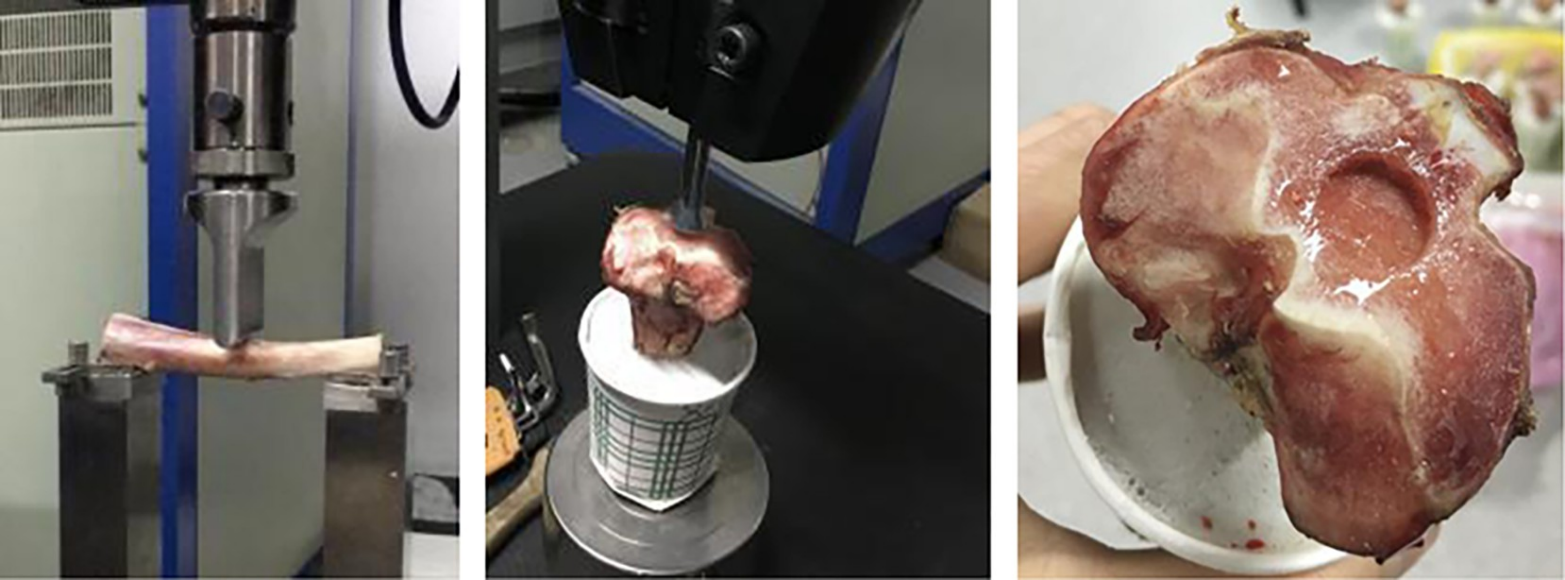
Mechanical testing. **A.** Three-point bending test. **B.** Indentation test. **C.** Proximal tibia after indentation test.

For the three-point bending test bones were cut both proximal and distal from the ablation site, leaving six cm on both sides (thereby guaranteeing equal length for all samples). Width and height of the sample were measured. The maximum differences for width and height from the average were only 1mm. Samples were placed on a hydraulic test machine (Model 64, MTS Systems, Eden Prairie, United States) with on both ends two cm supported by the machine. Subsequently force was gradually increased on the entry point from the needle with a blunt-shaped edge (see Fig 2A). Load deflection curve, maximal load (N) and displacement (mm) were measured. Testing was ended when the sample fractured. From these data stress and stiffness were determined. Stress is defined as the amount of force per mm^2^ (N/mm^2^) that the sample can bear before breaking. For calculating stress the following formula was used:

$$Stress=(3\;*\;Fmax\;*\;length)/(2\;*\;width\;*\;height^{2})$$

The shape of sheep tibia was in between spherical and rectangular. For our calculations we decided to consider the bones as rectangular. Stiffness was defined as the amount of force needed for one mm displacement (N/mm). Force was not reported since stress is a better predictor of strength in this test, accounting for the dimensions of the sample as well, where force does not hold this into account.

#### Indentation test

For the indentation test the bones were cut through the diaphysis making samples 13 cm high. The distal end was fixated in cement in a paper cup. Samples were kept in a fridge overnight, to prevent decrease in bone quality during the time the cement hardened. The next day indentation test was performed in the mechanical lab on a hydraulic test machine (Model 64, MTS Systems, Eden Prairie, United States). A bar with a 13mm diameter was used as indenter and fixed in the machine (see Fig 2B). Subsequently pressure was gradually built up. The machine measured the force needed per mm indentation into the bone (= displacement). Stress was calculated by applying the formula ‘test force (in N) / area of indentation (A)’. The area of indentation was a constant 132.73 mm^2^ (given the radius of the indenter was 6.5mm). Testing was ended when a sudden drop in force occurred, indicating fracture of the internal structure of the bone.

### Data analysis

Data was reported as mean value ± standard deviation (SD). Data were analyzed using SPSS statistics v25 (IBM, Armonk, United States). Given the relatively small sample size non-parametric testing was performed. Differences between ablation settings and controls were examined using a Mann-Whitney-U test. For all tests an alpha of .05 was chosen. For the in-vivo experiment no statistical testing was performed given the small group sizes.

## Results

### Ex-vivo

Values for maximum stress and stiffness in the three-point bending test are depicted in Table 1. The amount of stress needed to break the sample was significantly higher in the ablated samples compared to controls for the 60W - 10 min (p < .01) and 80W - 20 min (p = .015). The amount of stress needed to break the sample did not significantly differ between controls and 60W – 20 min (p = .093) and 80W - 10 min (p = .485). There was no difference in maximum stress between the four different settings. For stiffness no differences were found between the different settings (p > .05).

**Table 1:** Stress and stiffness (mean ± SD) in ex-vivo three-point bending test. * = significant.

Setting	Max stress (N/mm^2^)	Stiffness (N/mm)
60W, 10 min	118.51 (±4.8)*	1618.07 (±148.9)
60W, 20 min	108.94 (±12.7)	1288.17 (±169.3)
80W, 10 min	97.07 (±23.5)	1433.05 (±83.1)
80W, 20 min	112.40 (±12.5)*	1397.29 (±335.2)
Control	93.05 (±12.9)	1361.10 (±435.4)

**Fig 3:**
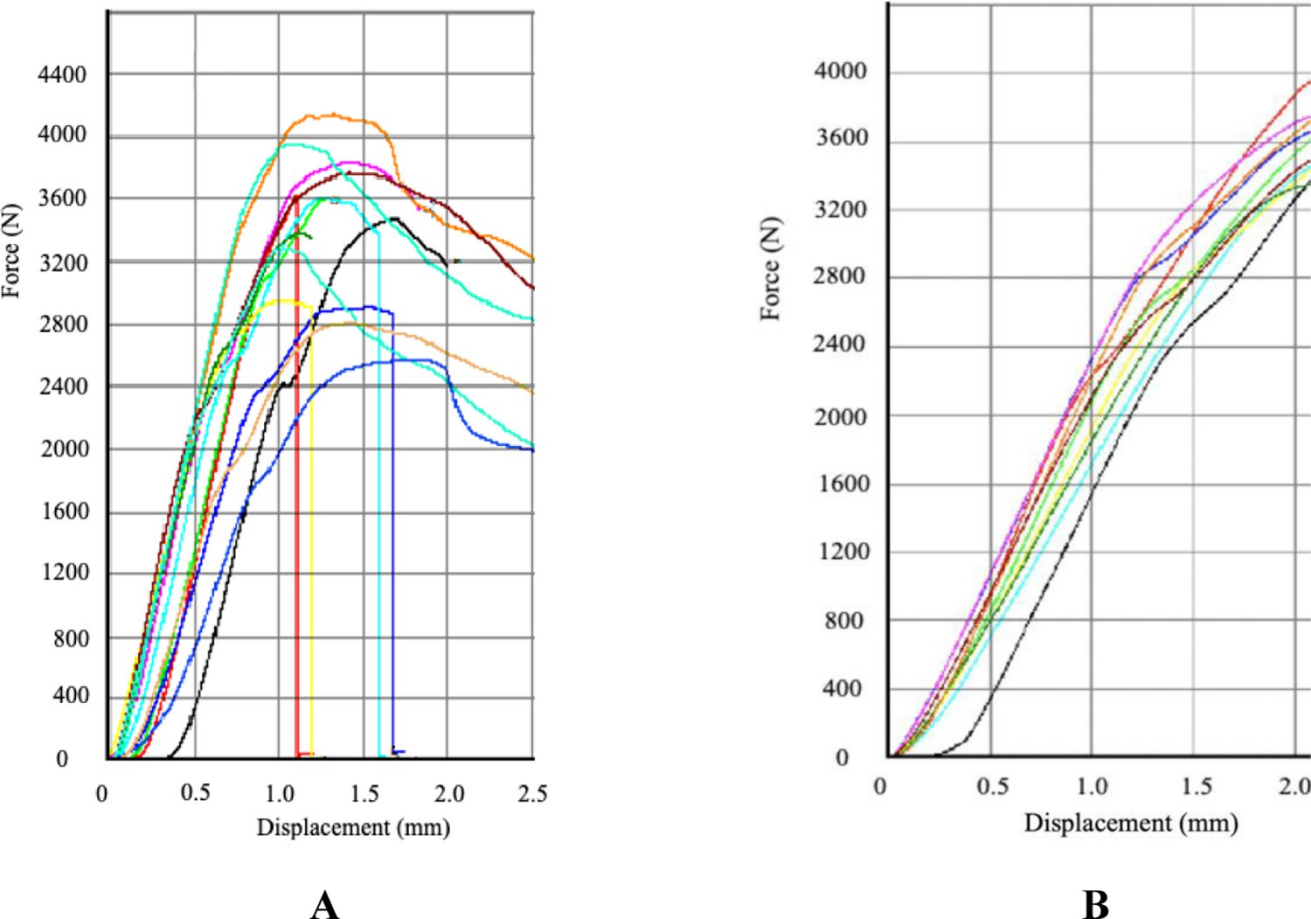
Load displacement curves of indentation test (A) and three-point bending test. Different lines represent individual samples. Figures only represent a limited number of samples.

Maximum force/maximum stress and stiffness as measured during the indentation test are depicted in Table 2. The force needed to break the control samples was significantly higher compared to the 60W - 10 min setting (p = .026). For 60W - 20 min (p = .132), 80W - 10 min (p = .065) and 80W - 20 min (p = .180) no significant difference was found despite the large difference in average values. Between the different settings no differences were found. For stiffness no differences were found between the different settings or between settings and controls (p > .05). Fig 3 depicts load displacement curves of both mechanical tests.

**Table 2:** Stress and stiffness (mean ± SD) in ex-vivo indentation test. * = significant.

Setting	Force (N)	Max stress (N/mm^2^)	Stiffness (N/mm)
60W, 10 min	981.79 (±285.2)*	7.40 (±2.1)	548.29 (±155.5)
60W, 20 min	1266.31 (±403.4)	9.54 (±3.0)	474.50 (±114.5)
80W, 10 min	1128.20 (±381.8)	8.50 (±2.9)	500.53 (±190.4)
80W, 20 min	1232.43 (±776.5)	9.29 (±5.8)	569.81 (±274.8)
Control	1809.59 (±737.9)	13.63 (±5.6)	470.69 (±244.1)

### In vivo

In two sheep in the in-vivo experiment a complication occurred. One (80W - 10 min, one week) had a fracture of the tibia (already before mechanical testing), the other one (60W - 10 min, six weeks) a deep infection. Both sheep were excluded from the analysis. In Table 3 the maximum stress and stiffness of the in vivo samples after one- and six weeks follow-up are depicted, as well as the average individual differences compared to the controls (contralateral tibia). Values for maximum stress and stiffness in the one week follow-up group were similar to the control tibias. Furthermore, no differences were found between a complete setting and the average control values after one week follow-up. However, after six weeks follow-up both maximum tolerable stress and stiffness for 60W – 20 min and stiffness for 80W – 10 min appear smaller than the control values. Stress for the 80W – 10 minute group did not differ from controls.

**Table 3:** Force, stress and stiffness of in-vivo indentation test (mean ± SD). Controls were measured in the contralateral tibia of the same animal.

Setting	Force (N)	Max stress (N/mm^2^)	Difference to control	Stiffness (N/mm)	Difference to control
**1 Week**					
60W, 10 min	818.89 (±199.0)	6.06 (±1.6)	- 0.12 (±3.6)	505.44 (±92.5)	+ 95.20 (±210.4)
60W, 20 min	1043.29 (±394.9)	7.86 (±3.0)	+ 0.08 (±0.6)	516.71 (±137.7)	- 162.99 (±293.3)
80W, 10 min	980.03 (±225.1)	7.38 (±1.7)	- 0.13 (±0.8)	691.37 (±225.6)	+ 170.42 (±213.0)
Control	945.78 (±285.9)	7.12 (±2.2)		538.43 (±235.5)	
**6 Weeks**					
60W, 10 min	1437.37 (±229.9)	10.83 (±1.7)	+ 1.57 (±4.0)	635.73 (±52.6)	+ 71.28 (213.5)
60W, 20 min	666.73 (±195.0)	5.02 (±1.5)	- 5.30 (±3.2)	279.52 (±127.2)	- 378.86 (±287.4)
80W, 10 min	891.29 (±384.7)	6.72 (±2.9)	- 3.42 (±2.8)	341.92 (±248.7)	- 449.70 (±307.7)
Control	1322.47 (±413.2)	9.96 (±3.1)		681.21 (±197.3)	

## Discussion

This is the first study to report both in- and ex-vivo mechanical effects of microwave ablation in long bone. Our results indicate no differences in mechanical properties ex-vivo and after one week follow-up for in-vivo ablations. After six weeks follow-up, a decrease in both stress and stiffness for the more aggressive settings was assumed.

Our results are in line with the findings of Herman et al [13], who found a 30% decrease in mechanical strength six weeks after MR-guided focused ultrasound ablation in pig ribs [13]. Yamamoto et al. [14] found no change in bone strength two months after RFA in rabbit femurs. Bucknor et al. [15] found subperiosteal new bone formation six weeks after MR-guided high-intensity focused ultrasound ablation. For MWA only ex-vivo data exists in literature. Ji et al. [16] found no change in mechanical strength in dog bone after microwave ablation (sacrifice intervals ranging from two weeks–one year). However, after sacrifice they kept the bones in a -80°C freezer for a non-documented time before examining mechanical strength [16]. This may have affected bone strength.

There was no decrease in stress and stiffness as a result of ablation in the three-point bending test. Therefore it was concluded that in an ex-vivo setting MWA did not influence immediate mechanical strength in the diaphysis. For 60W – 10 min and 80W – 20 min the stress (N/mm^2^) required to break the bone was even higher compared to controls. Due to limitations in the number of animals no testing was performed in-vivo and conclusions on three-point bending strength cannot be drawn from the present study for in-vivo applications. It seems likely that immediate strength is unaffected, but effects on mechanical strength after six weeks are not known and may very well be in line with the weakening seen in the metaphysis as a result of bone remodeling.

In the ex-vivo samples, the maximum force/stress needed to break the bone in the indentation test was only significantly lower for the 60W – 10 min ablations compared to controls. Average values were lower for all settings, though not significant. This is most likely due to the small number of samples and the relatively large standard deviation. After six weeks, stiffness appeared lower for the more aggressive settings (60W – 20 min, 80W – 10 min). Force/stress appears lower for these settings too. From these observations it can be concluded that there is no effect on mechanical strength one week after ablation. The decrease in strength found in the six week samples is probably the result of osteoblast/clast activity during remodeling. We hypothesize mechanical strength to increase again in the subsequent weeks as a result of increased osteoblast activity. It would be interesting to repeat the experiment with a 12-week follow-up group and larger group size to be able to perform statistical analysis on the in-vivo experiments.

Ji et al. [16] reported newly formed bone eight weeks after MWA. Bucknor et al. [15] reported new bone formation six weeks after high-intensity focused ultrasound ablation. This kind of biological rebuild can be expected from MWA as well, but cannot be confirmed in the present study. In the study of Ghomashchi et al. [17] histological effects of RFA were analyzed. They found no evidence of cortical thinning. Furthermore they demonstrated formation of newly formed trabeculae in the RFA zone with similar architecture, connectivity and mineral content compared to normal bone.

From the in-vivo indentation test data it was found that an average force of 1000N is needed to break the sample (for both one week and six weeks). This comes down to a mass of 100 kg on one leg (200 kg total body mass). Given an average human body mass of 80 kg, an average load up to 2.5 times the body mass is considered safe. Given the large variation in the data (for instance a force of 666.7N needed to break the sample six weeks after 60W – 20 min, corresponding to only an average load of 1.7 times the average mass) some caution in mobilization is recommended. However, these data support immediate mobilization after treatment. Since we removed the proximal cortical bone in the samples to create a flat surface for the indentation test, clinical mechanical strength will be even higher.

In the in-vivo experiment sheep were allowed to freely mobilize after treatment. Only in one out of 24 sheep a fracture occurred (= 4.2%). This corresponds to literature on minimally invasive treatment (fracture in 2.6–4.8% of cases) and curettage surgery (4.0%) and is lower than for wide resection (10.5%) [2, 5, 6].

The low number of fractures in minimally invasive treatment is potentially due to the fact that the integrity of the cortex is hardly affected by the technique.

One major study limitation is the small number of samples per group, especially in-vivo.

## Conclusion

Based on our initial results it appears that MWA decreases mechanical strength in bone to a small extent. However mechanical strength as measured in our study remains on a level sufficient to carry a patient’s mass. Therefore we think it is safe for patients to quickly start mobilizing again on the treated leg without significant risk of complications. However, since bone quality appears weaker after six weeks than after one week, caution is advised with the more aggressive settings. Further studies with larger sample sizes are required to confirm our findings.

## Supporting information

S1 Dataset (XLSX)
